# Design and validation of an exposure system for efficient inter-animal SARS-CoV-2 airborne transmission in Syrian hamsters

**DOI:** 10.1128/spectrum.04717-22

**Published:** 2023-10-26

**Authors:** Philip J. Kuehl, Justin Dearing, Adam Werts, Jason Cox, Hammad Irshad, Edward G. Barrett, Sean N. Tucker, Stephanie N. Langel

**Affiliations:** 1 Lovelace Biomedical Research Institute, Albuquerque, New Mexico, USA; 2 Vaxart, South San Francisco, California, USA; 3 Department of Pathology, Center for Global Health and Diseases, Case Western Reserve University School of Medicine, Cleveland, Ohio, USA; University of Mississippi Medical Center, Jackson, Mississippi, USA

**Keywords:** SARS-CoV-2, airborne transmission, transmission dynamics, Syrian hamster, golden hamster, aerosols

## Abstract

Severe acute respiratory syndrome coronavirus 2 (SARS-CoV-2) is a highly transmissible respiratory pathogen whose main transmission route is respiratory droplets and/or aerosols. The development of an animal model and exposure system that recapitulates airborne transmission of SARS-CoV-2 is integral to understanding the dynamics of SARS-CoV-2 spread in individuals and populations. Here, we designed, built, and characterized a hamster transmission caging and exposure system that allows for efficient SARS-CoV-2 airborne transmission from an infected index animal to naïve recipients under unidirectional airflow, without contribution from direct contact transmission. We hypothesized that a 1:1 or 1:4 ratio of infected index to naïve recipient hamsters would have comparable virological and clinical measurements after 8 h of exposure. Air concentrations and pulmonary deposited doses of SARS-CoV-2 in index and naïve hamsters, respectively, were statistically similar in both groups. Daily nasal viral RNA levels, terminal (day 5) lung viral RNA and infectious virus, and fecal viral RNA levels were statistically similar among 1:1 and 1:4 naïve animals. However, virological measurements in the 1:4 naïve animals were more variable than those in the 1:1 naïve animals, possibly due to hamster piling behavior creating uneven SARS-CoV-2 exposure during the grouped 1:4 airborne exposure. This resulted in slight, but not statistically significant, changes in daily body weights between the 1:1 and 1:4 naïve groups. Our report describes a multi-chamber caging and exposure system that allowed for efficient SARS-CoV-2 airborne transmission in single and grouped hamsters, regardless of ratio. This system can be used to better define transmission dynamics and test transmission-blocking therapeutic strategies against SARS-CoV-2 between individuals and among groups.

**IMPORTANCE:**

The main route of severe acute respiratory syndrome coronavirus 2 (SARS-CoV-2) transmission is airborne. However, there are few experimental systems that can assess the airborne transmission dynamics of SARS-CoV-2 *in vivo*. Here, we designed, built, and characterized a hamster transmission caging and exposure system that allows for efficient SARS-CoV-2 airborne transmission in Syrian hamsters without contributions from fomite or direct contact transmission. We successfully measured SARS-CoV-2 viral RNA in aerosols and demonstrated that SARS-CoV-2 is transmitted efficiently at either a 1:1 or 1:4 infected index to naïve recipient hamster ratio. This is meaningful as a 1:4 infected index to naïve hamster ratio would allow for simultaneous comparisons of various interventions in naïve animals to determine their susceptibility to infection by aerosol transmission of SARS-CoV-2. Our SARS-CoV-2 exposure system allows for testing viral airborne transmission dynamics and transmission-blocking therapeutic strategies against SARS-CoV-2 in Syrian hamsters.

## INTRODUCTION

Severe acute respiratory syndrome coronavirus 2 (SARS-CoV-2) is a highly transmissible respiratory pathogen spread by droplets and aerosols produced during coughing, sneezing, talking, and breathing (1 – 3). Airborne transmission of SARS-CoV-2 is dependent on multiple factors, including the duration and magnitude of viral shedding, the force of exhalation, the stability of the virus in aerosols and droplets, and the immune status of infected individuals (4, 5). SARS-CoV-2 can spread from an infected individual to one naïve individual or to a group of naïve individuals, particularly in congregate residency settings where residents have long-term and close contact (2). Deciphering the dynamics of SARS-CoV-2 transmission is an important goal for the development of public health strategies and biological countermeasures to inhibit transmission.

Animal models of SARS-CoV-2 transmission have been integral in defining the impact of SARS-CoV-2 replication, shedding, and immunity on viral transmission. The most common animal used for SARS-CoV-2 transmission studies is the Syrian golden hamster. Syrian hamsters are highly susceptible to SARS-CoV-2, recapitulate multiple aspects of the disease, and can transmit SARS-CoV-2 efficiently by aerosols, droplets, and fomites (6, 7). To study the airborne transmission of SARS-CoV-2 in Syrian hamsters, different chamber systems have been designed and implemented. For example, Port et al. and Dowall et al. used rodent cages connected via a connection tube that allowed airflow but no direct contact for studies assessing host and viral determinants of disease and interventions to decrease viral transmission (6, 8 – 12). In another report, four research groups reported on airborne transmission of SARS-CoV-2 BA.1 in Syrian hamsters using similar chamber systems but each with a different distance for the connecting tube (13). These groups have advanced the field of SARS-CoV-2 airborne transmission; however, there are still only a limited number of studies reporting the use of chamber systems to assess SARS-CoV-2 transmission, and only a small fraction of these assess viral levels in both animals and aerosols. The development and refinement of animal chamber systems are needed to better understand SARS-CoV-2 transmission dynamics.

Transmission dynamics exist between individuals (a single infected individual transmitting to a single naïve individual) or among groups (a single infected individual transmitting to a group of individuals). A direct comparison between these two SARS-CoV-2 transmission dynamics has not been assessed in a hamster model using an exposure system. Therefore, to study differences in transmission dynamics, we designed a chamber system where infected donor hamsters (index animals) are placed in an empty chamber (chamber 1) upstream of a chamber containing uninfected naïve hamsters (chamber 3). A connecting chamber (chamber 2) connects the index and recipient chambers without allowing physical contact between the hamsters while under constant unidirectional airflow controlled by a vacuum from chamber 3. The objective of our study was to compare SARS-CoV-2 transmission dynamics using an infected index hamster to one naïve recipient hamster (1:1) and an infected index hamster to a group of naïve recipient hamsters (1:4) in our caging and exposure system. Our hypothesis was that the 1:1 and 1:4 groups would have comparable virological and clinical measurements after 8 h of airborne exposure. We report the virological responses and clinical outcomes of index animals and naïve recipient animals (in either a 1:1 or 1:4 index to naïve ratio). Our data demonstrate that SARS-CoV-2 is transmitted efficiently from infected index to naïve recipient animals, regardless of the housing ratio, using our caging and exposure systems. While there are slight differences in viral replication kinetics, they do not result in significant differences between the naïve recipient treatment groups. Our data demonstrate that either a 1:1 or 1:4 ratio of index to naïve recipient Syrian hamsters can be used to study SARS-CoV-2 transmission dynamics. We also present the concentration of SARS-CoV-2 in the transmission aerosols and the calculated average deposited pulmonary dose of the naïve animals. Developing animal models and exposure systems for SARS-CoV-2 transmission is key to understanding the biology of virus-laden aerosols and for future testing of therapeutic strategies that limit airborne viral transmission.

## RESULTS

### Design and validation of a caging and exposure system for airborne transmission of SARS-CoV-2

**FIG 1:**
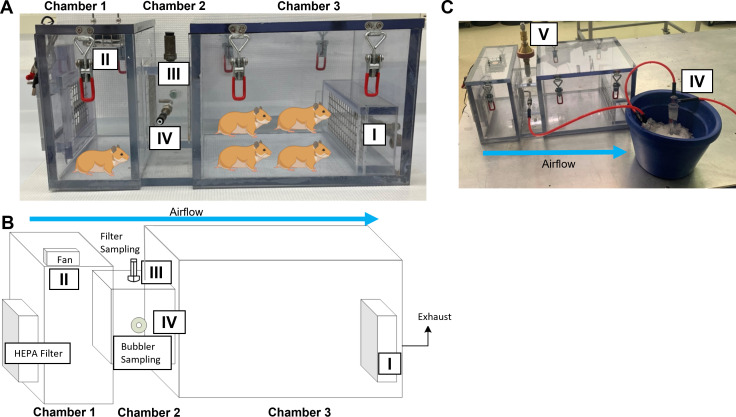
Three-chamber exposure system to detect SARS-CoV-2 RNA in the air after infecting index hamsters. (**A, B**) Chamber 1 housed the index hamsters, and chamber 3 housed the naïve hamsters. A wire mesh screen was fitted on either end of the connector chamber (chamber 2) to separate the hamsters but allow for air passage. Unidirectional airflow was controlled by house exhaust flow (I) from chamber 3, drawing room air into chamber 1 through a HEPA filter. A recirculating fan (II) was placed on top of chamber 1 to ensure the homogeneity of airborne particles moving through chambers 2 and 3. Aerosols for total aerosol concentration testing were collected using glass microfiber filters located at the top of chamber 2 (III, “filter sampling” in B). (**A,B,C**) Viral aerosol concentration was measured with an all-glass impinger connected to a probe port on the side of chamber 2 (IV, “bubbler sampling” in B). The particle size distribution was sampled with a portable aerosol spectrometer from either III or IV that was placed on top of chamber 2 (V).

**FIG 2:**
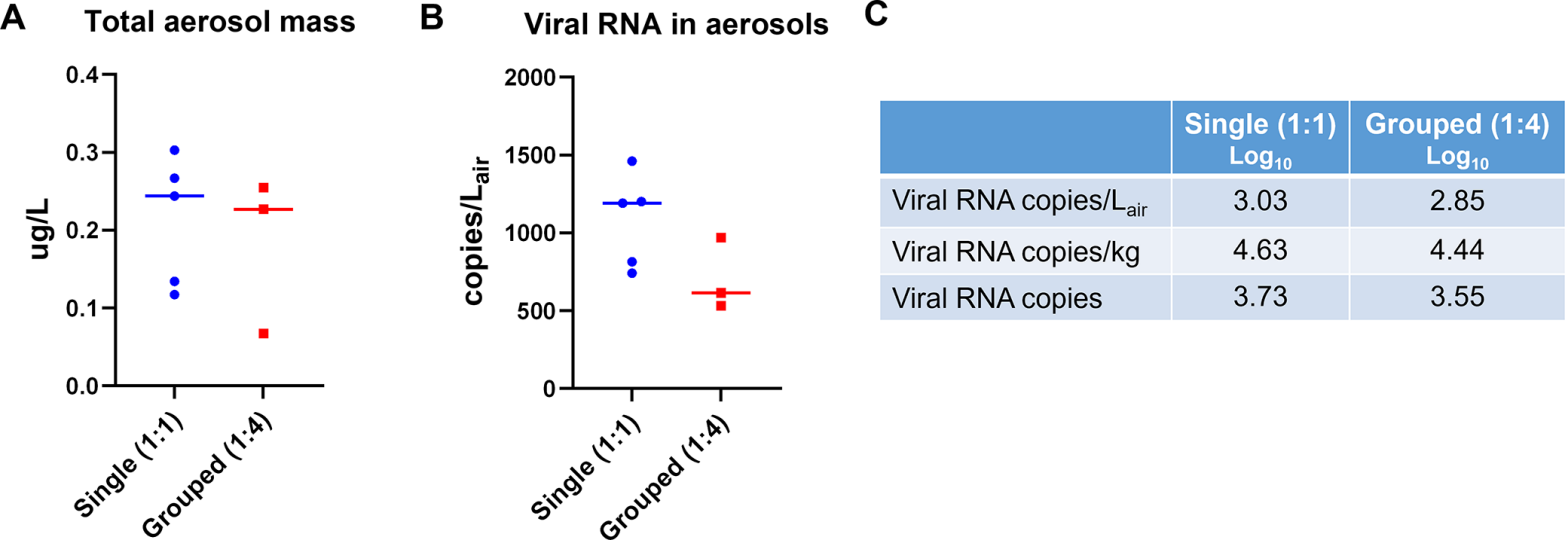
Total aerosol mass and viral RNA detected in aerosols in single (1:1) and group (1:4) hamsters. (**A**) Glass microfiber filters were used to collect aerosols, and total aerosol mass was measured by differential mass analysis using an ultra-balance (0.001 mg sensitivity). (**B**) Viral RNA in aerosols was collected into an all-glass impinger with Tris-EDTA buffer and measured via quantitative reverse transcription PCR. (**C**) Average SARS-CoV-2 viral RNA aerosols from single naïve (1:1) and group naïve (1:4) hamster chamber systems. Data were analyzed by an unpaired *t*-test.

Index hamsters were infected with 1 × 10^5^ TCID_50_ of SARS-CoV-2 by intranasal administration and housed individually. After 24 h, infected index hamsters were placed in chamber 1, and either one or four naïve recipient hamsters were placed in chamber 3 (Fig. 1A and B). Viral aerosol/droplet sampling was performed in the connector chamber (chamber 2) via glass microfiber filters (Fig. 1A and B) at 1 L/min for a total of 8 h. The total aerosol concentration of the filter samples determined by differential mass analysis resulted in an average concentration of 0.21 (±0.08) µg/L_air_ for single (1:1) and 0.18 (±0.1) µg/L_air_ for grouped (1:4) hamsters (Fig. 2A; data set S1 in supplemental material), which were not statistically different from each other. Additionally, particle size measurements were collected with a GRIMM aerosol spectrophotometer (Fig. 1C). Regardless of the duration of measurement or the number of collections taken, the number of aerosol particles collected was insufficient for the analysis of particle size. The low total aerosol concentration and failure to detect particle size likely reflect the low mass concentration of aerosol generated by the index hamster.

Aerosolized virus was collected in an all-glass impinger (Fig. 1A through C) filled with Tris-EDTA buffer downstream at a flow rate of 0.5 L/min for 8 h. The analysis of the impinger material (genomic viral RNA copies) showed an average viral RNA aerosol concentration of 1.08 × 10^3^ (±2.29 × 10^2^) copies/L_air_ for single (1:1) and 7.05 × 10^2^ (±2.33 × 10^2^) copies/L_air_ for group (1:4) hamsters (Fig. 2B; data set S2 in supplemental material). Utilizing standard methods (14) and a 10% deposition fraction, we calculated an average pulmonary deposited dose of 3.71 × 10^4^ copies/kg body weight or 4.74 × 10^3^ copies per animal in all groups. Specifically, the 1:1 hamster group’s average aerosol concentrations were 1.08 × 10^3^ copies/L_air_, 4.27 × 10^4^ copies/kg, or 5.43 × 10^3^ copies, and for the 1:4 animals, 7.05 × 10^2^ copies/L_air_, 2.78 × 10^4^ copies/kg, or 3.56 × 10^3^ copies (Fig. 2C; data set S3 in supplemental material). These data highlight the reproducibility of the aerosol concentration of SARS-CoV-2 viral RNA from the index animals.

### SARS-CoV-2-infected index hamsters transmit virus to single naïve (1:1) and group naïve (1:4) hamsters

**FIG 3:**
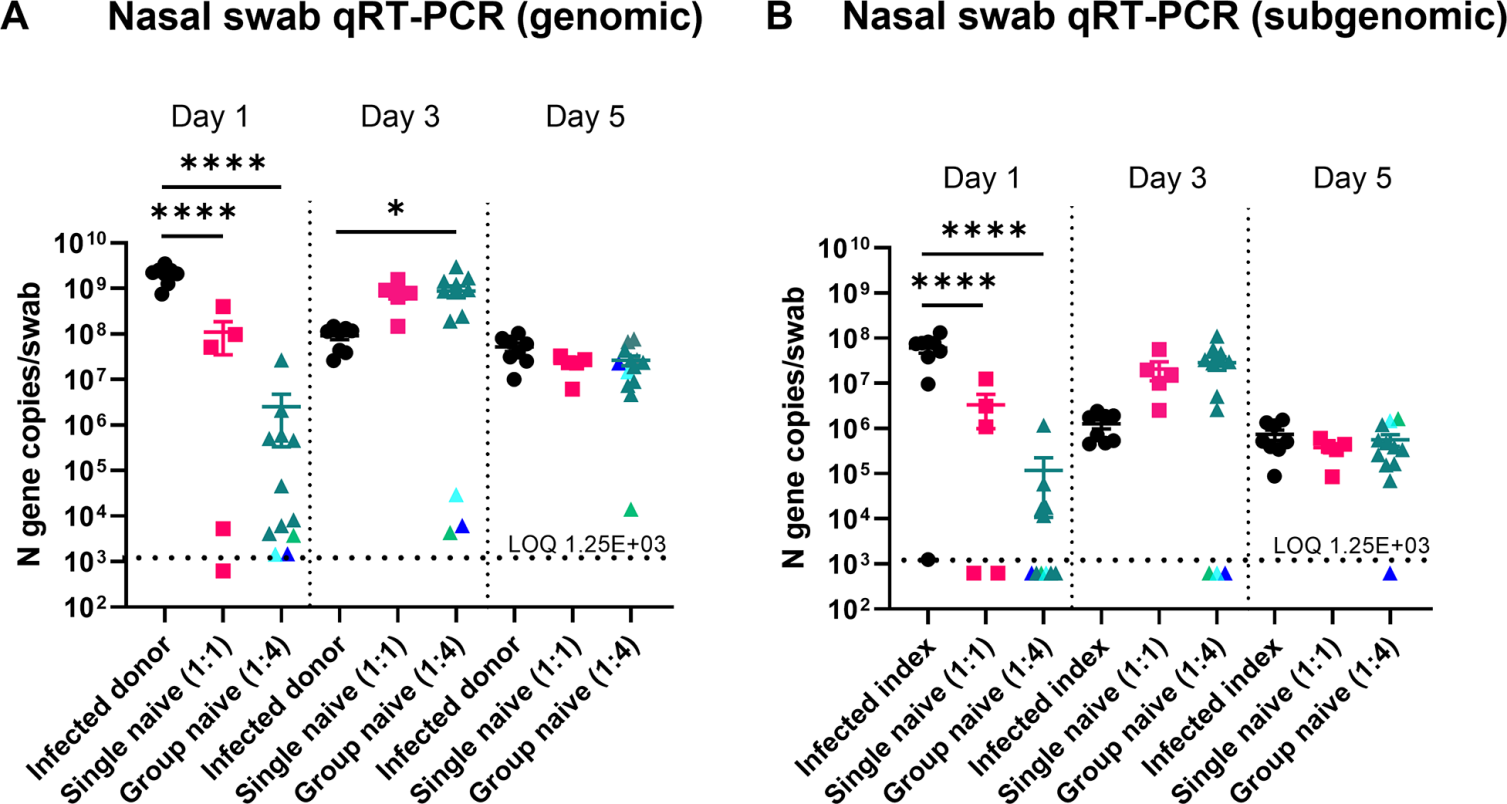
SARS-CoV-2 transmission and replication kinetics in infected index, single (1:1) naïve, and group (1:4) naïve recipient hamsters. Nasal swabs were collected on days 1, 3, and 5 post-inoculation in infected index hamsters and on days 1, 3, and 5 in naïve recipient hamsters after exposure to infected index hamsters in airborne transmission chambers. Viral genomic (**A**) and subgenomic (**B**) RNA levels were determined by quantitative reverse transcription PCR (qRT-PCR) of the nucleocapsid (N) gene. The different color triangles (turquoise, dark blue, green) for the group naïve (1:4) data points represent animals who remained near the limit of quantification (LOQ) at one or more time points (each animal retains the same color for each day post-SARS-CoV-2 exposure). Daily qRT-PCR data were analyzed by a one-way analysis of variance using Tukey’s multiple comparisons. Error bars represent the standard error of the mean. *P < 0.05, ****P < 0.0001.

Infected index hamsters reached peak levels of genomic (Fig. 3A) and subgenomic (Fig. 3B) viral RNA in nasal swabs at 1 d post-inoculation that decreased by day 5. For single (1:1) and group (1:4) naïve recipient hamsters, genomic and subgenomic viral RNA levels were detected in nasal swabs within 1 d post-exposure and reached peak levels on day 3 post-exposure. A higher proportion of the 1:4, but not 1:1, naïve recipient group hamsters had genomic and subgenomic viral RNA levels near the limit of quantification (LOQ) throughout the study. Despite this, genomic and subgenomic viral RNA levels in nasal swabs were not significantly different between 1:1 and 1:4 naïve recipient hamsters at any time point. Overall, this demonstrates that SARS-CoV-2 was transmitted effectively from infected index hamsters to single- and group-naïve recipient hamsters after 8 h of airborne exposure.

### Levels of lung viral RNA and infectious virus in group (1:4) naïve recipient hamsters

**FIG 4:**
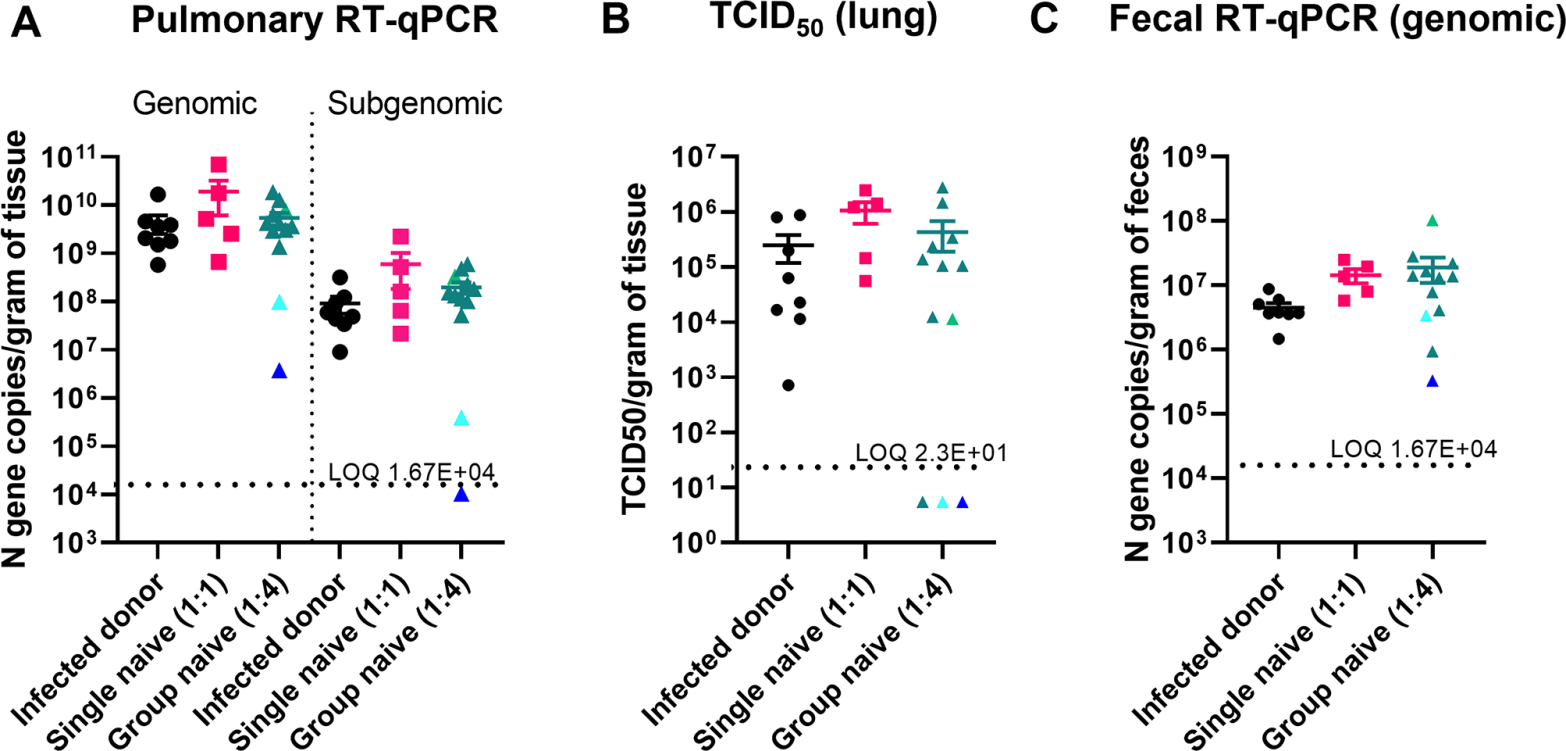
SARS-CoV-2 viral RNA levels in lung and feces between index, single (1:1) naïve, and group (1:4) naïve recipient hamsters at necropsy (day 5). Lung tissue (**A**) and feces (**C**) were collected at necropsy (day 5), and genomic (lung and feces) and subgenomic (feces only) RNA was isolated for SARS-CoV-2 detection by quantitative reverse transcription PCR of the nucleocapsid (N) gene. Infectious viral titers in lung tissue (**B**) were determined by TCID_50_. The dotted line represents the limit of quantification (LOQ). The different color triangles for the group naïve (1:4) data points represent animals who had lung subgenomic RNA and infectious viral titers near the LOQ (each animal retains the same color for each day post-SARS-CoV-2 exposure) or retained their respective color from Fig. 3. Data were analyzed by a one-way analysis of variance using Tukey’s multiple comparisons.

Lung genomic and subgenomic viral RNA (Fig. 4A) and infectious virus (Fig. 4B) were high in all groups and not significantly different between infected index, single naïve (1:1), and group naïve (1:4) hamsters at necropsy (day 5). Fecal viral genomic RNA levels (Fig. 4C), collected at necropsy (day 5), were also assessed and were not significantly different between infected index and naïve recipient groups. However, a greater proportion of the 1:4 naïve hamsters had lung viral RNA and infectious virus levels near the LOQ compared to the 1:1 naïve hamsters.

### Body weights and lung weights are similar between single (1:1) and group (1:4) naïve recipient hamsters

**FIG 5:**
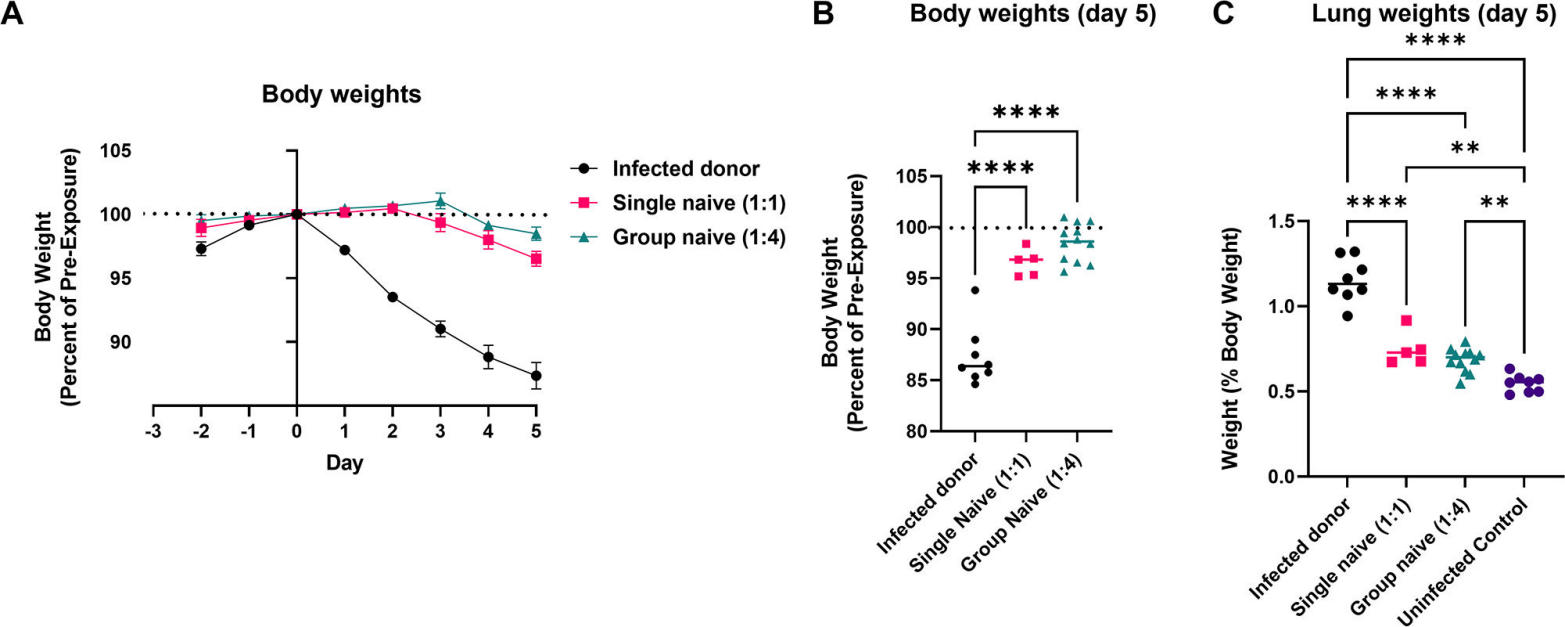
Clinical outcomes were similar between single (1:1) and group (1:1) naïve recipient hamsters. Daily weight changes (**A**) and terminal body weights (**B**) were determined by the percent of day 0 weight (relative to SARS-CoV-2 exposure). Terminal lung weights (**C**) were determined at necropsy. In B and C, data were analyzed by a one-way analysis of variance using Tukey’s multiple comparisons, respectively. Error bars in A represent the standard error of the mean. **P < 0.01, ****P < 0.0001.

Body weights as a percent of pre-SARS-CoV-2 exposure day 0 were measured in all groups. Infected index animals lost significantly more weight over the course of the experiment and had significantly lower terminal body weights (Fig. 5A and B). Body weights over time and at necropsy were not significantly different between single (1:1) and group (1:4) naïve recipient hamsters.

Body weight-normalized lung weights, an indirect measure of pulmonary inflammation, were compared among all groups, including uninfected control hamsters (Fig. 5C). All infected groups had significantly higher lung weights than uninfected control hamsters. However, lung weights were highest in the infected index hamsters. There were no significant differences in lung weights between 1:1 or 1:4 naïve hamsters.

## DISCUSSION

Considering the high heterogeneity in SARS-CoV-2 transmission dynamics observed in humans (15, 16), stringently controlled experimental settings to study host and viral factors of SARS-CoV-2 transmission are needed. SARS-CoV-2 transmission dynamics play a significant role in the ongoing coronavirus disease 2019 pandemic. Animal models of SARS-CoV-2 transmission allow for the study of environmental and host factors that impact viral transmission and replication, as well as the testing of therapeutic strategies (17). However, viral transmission characteristics differ for aerosols and droplets compared to direct contact or fomite exposure (6). Therefore, designing caging and exposure systems that allow for airborne transmission of SARS-CoV-2 in animals without contributions from other routes of exposure is important to understand transmission dynamics and test therapeutic strategies that inhibit airborne transmission. Here, we designed and characterized a caging and exposure system for SARS-CoV-2 airborne transmission and validated it using infected index hamsters cohoused but physically separated from either one (single) or four (grouped) naïve recipient hamsters.

Previous groups have demonstrated that SARS-CoV-2 is transmitted efficiently from infected index to naïve recipient hamsters. Sia and colleagues infected index hamsters with 8 × 10^4^ TCID_50_ of SARS-CoV-2 and, after 24 h, placed each index animal in a new cage cohoused with one naïve hamster (7). SARS-CoV-2 was detected in cohoused hamsters at 1 d post-cohousing and reached peak viral loads in nasal washes at day 3. However, this experiment could not distinguish between airborne and fomite transmission considering that naïve hamsters were cohoused in a way that allowed contact with infected index hamsters. In more recent reports, Port and colleagues designed a chamber system where SARS-CoV-2 transmission was observed under constant airflow of airborne particles <5 µm over a 200-cm distance (18). Indeed, SARS-CoV-2 transmission could occur at this distance between Syrian hamsters even within 1 h at a 2:2 ratio (two infected index and two recipient naïve hamsters). Investigators were also able to demonstrate with this system that airborne transmission dynamics and aerosol characteristics (viral load and particle size) were different between SARS-CoV-2 variants and male and female hamsters (10, 18). Dowall et al. used a set of three cages, each linked through a 130-mm-diameter connector, and reported SARS-CoV-2 RNA shedding in donor and recipient animals. However, they did not quantify RNA levels in aerosols or droplets. In another recent report, four independent research groups determined that SARS-CoV-2 BA.1 had reduced airborne transmission using similar chamber systems but with variable distances of the connecting tube, exposure times, and inoculum amounts (13) but similarly did not report virological data from aerosols.

In our study, we collected airborne particles using glass impingers, quantified SARS-CoV-2 viral RNA, and calculated an average deposited pulmonary dose for the naïve animals. We observed similar amounts of SARS-CoV-2 viral RNA in airborne particles between chambers of the 1:1 and 1:4 treatment groups. This suggets that index animals inoculated with a consistent dose of SARS-CoV-2 (1 × 10^5^ TCID_50_/animal) transmitted similar amounts of SARS-CoV-2 in aerosols to naïve hamsters in our unidirectional airflow exposure system. At day 1 post-exposure, nearly all the naïve hamsters in the 1:1 and 1:4 groups had viral RNA levels above the LOQ. Peak genomic and subgenomic viral RNA levels were observed on day 1 for the index hamsters and day 3 for the naïve recipient group. This could be due to a lower inoculation dose in the naïve recipient compared to index groups or that viral exposure was delayed in the naïve recipient group as infectious SARS-CoV-2 in the air can last up to 4 d post-infection (10). Interestingly, in the 1:4 naïve recipient group, there were a greater proportion of hamsters on days 3 (16.67%) and 5 (8.3%) that had nasal swab viral RNA levels that remained near the LOQ compared to 0% in the 1:1 naïve recipient group. This demonstrates a moderate increase in variability of SARS-CoV-2 exposure and/or replication in the 1:4 naïve compared to the 1:1 naïve recipient hamsters. Hamsters are known to group together when cohoused and were observed to be sleeping in piles in some transmission chambers during the exposure period. It is possible that the variability observed was due to hamster(s) piled underneath the other hamsters during cohousing with the infected index hamsters and therefore did not receive a similar inoculum dose via aerosols or droplets as its cage mates. Despite this, statistically, there were no differences in nasal swab viral RNA levels between the 1:1 or 1:4 naïve treatment groups.

Like virological measurements in nasal swabs, lung viral RNA levels, lung infectious viral titers, and fecal viral RNA levels were more variable in the 1:4 than in the 1:1 naïve recipient group. The explanation is expected to be similar to the nasal swabs: hamsters piling together may lead to an uneven distribution of transmitted SARS-CoV-2 during airborne exposure from infected index hamsters. Additionally, the mean body weights (as a percent of pre-exposure) were numerically higher in 1:4 than in 1:1 naïve recipients. This suggests that the potential lower SARS-CoV-2 exposure in some of the 1:4 hamsters resulted in less body weight loss over the course of the experiment. However, for both virological measurements and clinical signs, the overall levels were not significantly different between the 1:1 and 1:4 naïve recipient groups. Therefore, it is unlikely that the variability observed in the 1:4 naïve recipient groups will significantly impact a study’s design and/or outcomes, depending on the research question. Additionally, we collected nasal swabs and whole lungs to determine virological parameters in our study animals. Other groups have also tested nasal washes (13) and oral swabs (10, 13) for RNA and infectious viral titers. Different sampling methods in either the nasal or oral cavity may impact reported RNA or infectious titers. This should be considered when comparing virological assessments between studies.

There are a few limitations to our study. For example, we cannot rule out dander or other small fomites transfer from donor to naïve animals, among other naïve animals or the chamber itself. Additionally, our system does not discriminate between aerosols and large droplets, so our animals are likely receiving a mixture of both. We were not able to determine particle size, likely due to the low mass concentration produced by the index hamsters. While other groups have presented particle size distributions from transmission systems (10, 19), a different instrumentation was used compared to our study, as well as nonstandard data reporting methods. Additionally, modifications to these systems (anesthetizing animals for collection and assessment of particle size data, not correcting for background particles from healthy animals, etc.) do not allow for direct comparisons to the data we present here. Based on the low concentration of SARS-CoV-2 in the aerosol, the only means to differentiate the size is via settling velocity, as was done by Port et al. (8). Harmonizing methodology across research groups engaged in SARS-CoV-2 transmission research is a critical improvement needed for the field. Lastly, it is possible that naïve animals transmit viruses to each other, contributing to transmission dynamics.

Developing animal models and exposure systems to study SARS-CoV-2 transmission is integral to defining transmission kinetics and developing therapeutics that either prevent or stop viral transmission to naïve individuals. Here, we designed a caging and exposure system and demonstrated that SARS-CoV-2 is transmitted efficiently at either a 1:1 or 1:4 infected index to naïve recipient hamster ratio. From a practical standpoint, using a 1:1 ratio would require a large number of chambers and might not be feasible for comparing interventions effectively. A 1:4 ratio would allow comparisons of interventions in multiple naïve animals, allowing genetic and biological variability to contribute to transmission dynamics. In conclusion, we designed, built, and validated an exposure system that can be used to better define viral airborne transmission dynamics and to test transmission-blocking therapeutic strategies against SARS-CoV-2.

## MATERIALS AND METHODS

### Characteristics of the transmission chamber system

The exposure system allowed unidirectional air flow from SARS-CoV-2 (USA WA1/2020) infected index hamsters in the first chamber (4″ × 10″ × 9″) to naïve recipient hamsters in the third chamber (10″ × 10″ × 9″). A second chamber (4″ × 5″ × 5″) connected chambers 1 and 3 and was raised 1 inch off the ground to prevent feces and large fomite transfer. The chambers were made of polycarbonate. Wire mesh screens (0.25″ × 0.25″ holes) were fit on either ends of the connector chamber to separate the hamsters but allow for air passage. The first chamber was fitted with a recirculating fan to ensure the homogeneity of airborne particles moving through chambers 2 and 3. The fan had a wire cage around it to prevent animal access. Unidirectional flow was controlled by house exhaust flow from chamber 3 at 5 L/min, drawing room air into chamber 1 through a HEPA filter (Kenmore EF-2 HEPA Filter 86880).

Sampling was performed in the connection chamber via probe ports. Filter samples (glass microfiber filters, Whatman Type GF/A) located at the port on top of chamber 2 were collected at 1 L/min to determine the total aerosol concentration. Filters were collected for between 1 and 8 h to collect sufficient material for differential mass analysis via ultra-balance (0.001 mg sensitivity). Particle size distribution was sampled with a GRIMM portable aerosol spectrometer (Model 1.109, GRIMM Technologies, Inc., Germany). Viral aerosol concentration was measured with an all-glass impinger (Midget Impinger, ACE Glass Inc.) located at the port on the side of chamber 2. Impingers were filled with 10 mL of TE buffer and collected at a flow rate of 0.5 L/min for up to 8 h. The remaining TE buffer was weighed (to normalize for evaporation during collection) and assayed for SARS-CoV-2 by quantitative reverse transcription PCR (qRT-PCR).

### Animals

Male Syrian hamsters (*Mesocricetus auratus*) 12 to 14 weeks of age (weight range 106 to 136 g) were sourced from Charles River Laboratory. Animal work was performed at Lovelace Biomedical with approval from the Institutional Animal Care and Use Committee. Animals were received and quarantined outside of the ABSL3 containment facility for at least 1 week prior to moving into the ABSL3, where all procedures were performed. Hamsters were individually housed in filter-topped cage systems and supplied with a certified diet, filtered municipal water, and dietary and environmental enrichment prior to transmission experiments. Standard environmental conditions were maintained throughout quarantine and study procedures (temperature 18°C–26°C, relative humidity 30%–70%, 12 h alternating light/dark cycles, and water *ad libitum*).

Index animals were anesthetized by an intraperitoneal injection of 10 mg/kg ketamine and 5 mg/kg xylazine cocktail for inoculation. Vaporized inhalant isoflurane gas (maximum of 5%) was used if additional restraint was necessary. Upon reaching the desired plane of anesthesia, the animal was held upright and intranasally inoculated dropwise with 100 µL per naris of a 5 × 10^5^ TCID_50_/mL SARS-CoV-2 in Dulbecco’s Modified Eagle’s Medium inoculum for a total of approximately 1 × 10^5^ TCID_50_/animal. All infected animals were monitored cage-side until their righting reflex was restored.

Approximately 24 h later, the transmission chambers were prepared, and animals were loaded at either a 1:1 ratio (five experimental repeats) or a 1:4 ratio (three experimental repeats) of infected index hamsters to naïve recipient hamsters. During the 8-h transmission experiment, bedding and enrichment were removed; however, DietGel Boost (Clear H_2_O, product code: 72-04-5022) was provided *ad libitum* in both chambers 1 and 3 to meet the food and water needs of the animals throughout their 8 h duration in the chamber. Naïve recipient hamsters were loaded and secured in chamber 3 before the infected index animals were handled. Animals were observed regularly while on exposure to monitor welfare. At the end of the 8-h exposure, naïve hamsters were returned to their home cages before the infected index animals were handled. Each time animals were handled, technicians put on new personal protective equipment to reduce the risk of SARS-CoV-2 transfer from handling infected index animals.

Nasal swabs were collected with Maxapplicators (Plasdent) with 0.5-mm ultra-fine fiber head tips. The animal was briefly anesthetized with vaporized inhalant isoflurane anesthetic, and the swab was inserted 2–4 mm into either the right or left naris and circled or twisted gently. The swab was then removed, placed into a Safe-Lock Eppendorf tube containing 1 mL of Trizol (TRI reagent, Millipore Sigma), and cut at the hinge point. Swabs were flash frozen at −80°C ± 10°C for future RNA isolation. The nares sampled were alternated at each time point.

Animals were euthanized via intraperitoneal injection with a barbiturate overdose of pentobarbital (390 mg/mL) diluted in 1:10 in normal saline. After confirmation of death, the lungs were removed, weighed, and placed into a Safe-Lock Eppendorf tube and flash frozen at −80°C ± 10°C for future RNA isolation and virus quantification by TCID_50_. Additionally, 2–3 fecal pellets were collected from the rectum, weighed, and placed into a Safe-Lock Eppendorf tube and flash frozen at −80°C ± 10°C for future RNA isolation.

### RNA isolation of nasal swabs, lung samples, and feces

Lung tissue and feces samples were weighted (≤75 mg) and homogenized with beads using a Tissue Lyzer (Qiagen) in 1 mL of TRI reagent before RNA was isolated and purified from tissue samples using the Direct-Zol 96-RNA Kit (Zyma Research). Nasal swabs were removed from −80°C ± 10°C storage and mixed with TRI reagent (between 0.3 and 1 mL). Samples were mixed by vortexing briefly and allowed to incubate at room temperature for 15 min prior to RNA isolation.

### Detection of SARS-CoV-2 genomic and subgenomic RNA by qRT-PCR

Copies of the SARS-CoV-2 N gene (genomic) were measured by qRT-PCR TaqMan Fast Virus 1-step assay (Applied Biosystems). SARS-CoV-2-specific primers and probes from the 2019-nCoV RUO Assay Kit (Integrated DNA Technologies) were used: (L primer: TTACAAACATTGGCCGCAAA; R primer: GCGCGACATTCCGAAGAA; probe: 6FAM-ACAATTTGCCCCCAGCGCTTCAG-BHQ-1). Reactions were carried out on a Stratagene MX3005P or BioRad CFX384 Touch instrument according to the manufacturer’s specifications. A semi-logarithmic standard curve of synthesized SARS-CoV-2 N gene RNA (LBRI) was obtained by plotting the Ct values against the logarithm of cDNA concentration and used to calculate the SARS-CoV-2 N gene in copies per gram of tissue. Copies of the SARS-CoV-2 E gene (subgenomic) were measured by qRT-PCR with the TaqMan Fast Virus 1-step assay (Thermo Fisher). SARS-CoV-2-specific primers and probes: sgLeadSARSCoV2-F 5′-CGATCTCTTGTAGATCTGTTCTC-3′; E Sarbeco R: 5′-ATATTGCAGCAGTACGCACACA-3′; E_Sarbeco_P1: 6FAM-ACACTAGCCATCCTTACTGCGCTTCG-BHQ-1; and E Sarbeco F: 5′-ACAGGTACGTTAATAGTTAATAGCGT-3′. Reactions were carried out on a Stratagene MX3005P or BioRad CFX384 Touch instrument according to the manufacturer’s specifications. A semi-logarithmic standard curve of synthesized SARS-CoV-2 E gene RNA (LBRI) was obtained by plotting the Ct values against the logarithm of cDNA concentration and used to calculate the SARS-CoV-2 N gene in copies per gram of tissue.

### Statistical analysis and aerosol concentration calculations

The methods used for determining significance were a one-way analysis of variance and Tukey’s multiple comparisons using GraphPad Prism Software.

The aerosol dose for the naïve hamsters was calculated using an inhaled dose equation (14):


Dose(mg/kg/d)=(C(copies/L)×RMV(L/min)×D(min)×DF/)BWT(kg).

where *C* is the concentration (copies/L) in air inhaled (aerosol concentration); RMV, respiratory minute volume (L/min); *D*, duration of exposure (min); DF, deposition fraction; BWT, body weight (kg). For example, the aerosol concentration was the average aerosol concentration (copies/L_air_), the exposure duration (480 min), the average group body weight (1:1 and 1:4 groups), and the assumed deposition fraction of 10%. While there are no specific data for a 10% deposition fraction for hamsters, it is likely that the deposition will be like that used for rats and mice.

Particle size distribution samples all showed insufficient particle collections (background levels) across the entire particle size range for the GRIMM instrument. Therefore, no particle size reduction or analysis was performed.

## SUPPLEMENTAL MATERIAL

Supplemental Material spectrum.04717-22-s0001.xlsx Supplemental Table 1–3

ASM does not own the copyrights to Supplemental Material that may be linked to, or accessed through, an article. The authors have granted ASM a non-exclusive, world-wide license to publish the Supplemental Material files. Please contact the corresponding author directly for reuse.
